# Down-regulation of ABCE1 inhibits temozolomide resistance in glioma through the PI3K/Akt/NF-κB signaling pathway

**DOI:** 10.1042/BSR20181711

**Published:** 2018-12-11

**Authors:** Peng Zhang, Xiao-Bing Chen, Bing-Qian Ding, Hong-Lin Liu, Tao He

**Affiliations:** Department of Neurosurgery, Huaihe Hospital of Henan University, Kaifeng 475000, China

**Keywords:** ATP binding cassette E1 (ABCE1), glioma, PI3K/Akt/NF-κB signaling pathway, temozolomide (TMZ) resistance

## Abstract

The ATP binding cassette (ABC) E1 (ABCE1), a member of the ABC family, was originally described as the RNase L inhibitor. Through forming a heterodimer with RNase L, ABCE1 participates in the negative regulation of the 2-5A/RNase L system and thus mediates a wide range of biological functions. Recent evidence has shown the new roles of ABCE1 in tumorigenesis. However, there have been no investigations on the specific effect of ABCE1 on glioma. In the present study, we examined the expression pattern and possible role of ABCE1 in glioma. Our study demonstrated that ABCE1 was up-regulated in glioma tissues and cell lines. Down-regulation of ABCE1 inhibited temozolomide (TMZ) resistance of glioma cells *in vitro* and *in vivo*. In addition, we found that the PI3K/Akt/NF-κB pathway was involved in ABCE1-mediated chemoresistance of glioma cells. Taken together, our study suggested ABCE1 as a promising target for glioma chemotherapy.

## Introduction

Glioma, a common type of brain tumor, accounts for ∼30% of cancers in the central nervous system [1,2]. Although many therapeutic approaches for glioma have been improved, the treatment effect is unsatisfactory and the median survival time of glioma patients is still less than 12 months [3,4]. This dismal situation desperately demands a variety of novel therapies. Numerous clinical practices have demonstrated that temozolomide (TMZ), a DNA-alkylating antineoplastic drug, is greatly useful in delaying tumor progression and extending patient survival, thus TMZ has been adopted as the first-line treatment for glioma [5–7]. But recent studies showed that the therapeutic efficiency of TMZ is limited due to chemoresistance in tumors, which has become a serious impediment in glioma treatment [8–12]. Therefore, it is urgently needed to better understand the mechanisms underlying TMZ resistance in order to improve the poor outcome of glioma patients.

The ATP binding cassette (ABC) family contains a group of transporters performing various functions in cells [13,14]. So far, a total of 49 members have been identified in the family [15]. These members are further divided into seven subfamilies designated as ABC A–G [16]. Recently, a growing body of evidence has indicated that ABC E1 (ABCE1), a member of the ABC family, is associated with some biological behaviors of cancer cells, such as proliferation, migration, and invasion [17,18]. Furthermore, ABCE1 has been reported to be abnormally expressed in a variety of drug-resistant cell types, suggesting its certain effect on chemoresistance [19]. However, there have been no investigations on the specific role of ABCE1 in glioma.

In the present study, we examined the expression pattern of ABCE1 in glioma and tested the effect of ABCE1 knockdown on glioma sensitivity to TMZ. Our study demonstrated that ABCE1 was up-regulated in glioma tissues and cell lines. Down-regulation of ABCE1 inhibited TMZ resistance of glioma cells *in vitro* and *in vivo*. In addition, we found that the PI3K/Akt/NF-κB pathway was involved in ABCE1-mediated chemoresistance of glioma cells. Taken together, our study suggested ABCE1 as a promising target for glioma chemotherapy.

## Materials and methods

### Patients and tissue samples

Human glioma tissues and corresponding normal brain tissues were obtained from 38 patients at the Huaihe Hospital of Henan University (Kaifeng, China). The study was performed with written consents from each patient and the approval of the Ethics Committee of Henan University. All tissue samples were collected and then frozen in liquid nitrogen and stored at −80°C for future use.

### Cell lines and cell culture

Human glioma cell lines (U87 and A172) and normal human astrocyte (NHA) were purchased from the American Type Culture Collection (ATCC, Manassas, VA, U.S.A.). All cell lines were cultured in RPMI-1640 (Sigma, St. Louis, MO, U.S.A.) containing 10% FBS (Sigma), 100 mg/ml penicillin, and 100 μg/ml streptomycin, followed by incubation at 37°C in a humidified atmosphere with 5% CO_2_.

### Quantitative real-time PCR

Total RNA was extracted from tissues or cells using TRIzol reagent (Invitrogen, Carlsbad, CA, U.S.A.) and reversely transcribed into cDNA using the PrimeScript RT Reagent Kit (Takara Biotechnology, Dalian, China). RT-PCR was performed on an ABI PRISM 7300 thermocycler (Applied Biosystems, Foster City, CA, U.S.A.). The reaction conditions were 96°C for 1 min, 40 cycles of 96°C for 15 s, and 72°C for 30 s. The following primers were used: ABCE1, 5′-TTGGTTGTGGGAAGTCGT-3′ (forward) and 5′-GCTTATGTAGTTAATGGGAGGT-3′ (reverse); GAPDH, 5′-GAGTCAACGGATTGGTCGT-3′ (forward) and 5′-GACAAGCTTCCCGTTCTCAG-3′ (reverse). The relative expression levels were normalized to GAPDH and determined using the 2^−ΔΔ*C*^_t_ method.

### Western blot analysis

Tissues or cells were lysed in lysis buffer (Gibco, Rockville, MD, U.S.A.). Protein concentration was determined using a BCA Protein Assay Kit (Pierce, Rockford, IL, U.S.A.). An equal amount of protein was separated with 12% SDS/PAGE and then transferred on to PVDF membranes (Millipore, Billerica, MA, U.S.A.). After blocking in 5% skim milk, the membranes were subjected to overnight incubation at 4°C with primary antibodies against ABCE1, p-PI3K, PI3K, p-Akt, Akt, NF-κB, and GAPDH. Subsequently, the membranes were washed with TBST and then incubated with appropriate secondary antibody for 1 h at room temperature. All antibodies were purchased from Santa Cruz Biotechnology (Santa Cruz, CA, U.S.A.). Protein bands were visualized using an ECL detection kit (Pierce) and their density was analyzed using the ImageJ software.

### Cell transfection

For ABCE1, NF-κB/p65 or Akt knockdown, cells were transfected with corresponding shRNAs (OriGene, Rockville, MD, U.S.A.) using Lipofectamine 2000 (Invitrogen) according to the manufacturer’s instructions. The shRNA sequences were as follows: 5′-GCTACAGCGAGTACGTTTACCT-3′ for ABCE1 and 5′-GAGACCTCAGTATGTTACCTGT-3′ for negative control; 5′-GAUGAGAUCUUCCUACUGUdTdT-3′ for NF-κB/p65 and 5′-UUCUCCGAACGUGUCACGUTTdTdT-3′ for negative control; 5′-UGCCCUUCUACAACCAGGATT-3′ for Akt and 5′-UCCGUUUCGGUCCACAUUCTT-3′ for negative control. Forty-eight hours after transfection, the knockdown efficiency was confirmed by Western blot analysis.

### Cell viability assay

Cell viability was measured via the CCK-8 assay. Cells were seeded in a 96-well plate at a density of 2 × 10^3^ cells/well and cultured for 24 h. After treatment with different concentrations of TMZ, cells were cultured for 48 h and then CCK-8 solution (Dojindo, Japan) was added. Cell viability was detected at 450 nm using a microplate reader. The survival of control cells was set at 100% and used to calculate IC_50_ as previously described [20].

### Flow cytometry

Cell apoptosis was detected using an Annexin V-FITC apoptosis detection kit (eBioscience, Waltham, MA, U.S.A.). In brief, cells were treated with TMZ (100 μM) for 24 h and then resuspended in binding buffer containing annexin V and PI. After incubation in the dark for 15 min, the apoptotic rate was analyzed on a FACSCalibur instrument (BD Bioscience, San Diego, CA, U.S.A.).

### *In vivo* xenograft tumor assay

Male nude mice (4–5 weeks old) were obtained from Shanghai Laboratory Animal Center. All animal experiments were approved by the Animal Care and Use Committee of Henan University. Transfected cells (5 × 10^6^) were subcutaneously injected into mice. When tumors reached ∼150 mm^3^, mice were intraperitoneally injected with 50 mg/kg TMZ everyday. Tumor size was measured every 5 days. Tumor volume was calculated by the following formula: V = length × width^2^ × 0.5. Thirty days later, mice were killed and tumors were stripped.

### Statistical analysis

Data were expressed as means ± S.D. Statistical analysis was performed using SPSS 17.0 software. Student’s *t*test or one-way ANOVA was used to compare the differences between different groups. *P*<0.05 was considered statistically significant.

## Results

### Expression of ABCE1 is elevated in glioma tissues and cell lines

**Figure 1:**
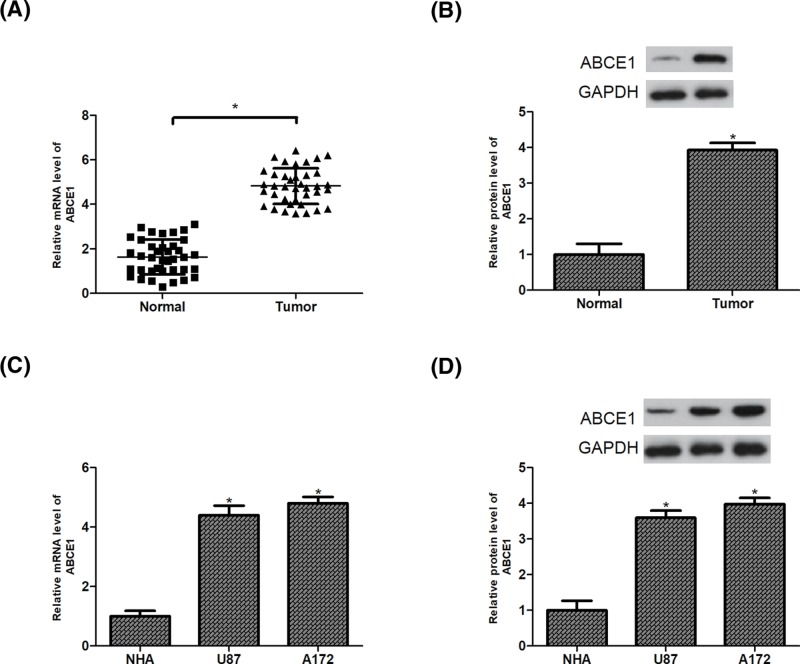
Expression of ABCE1 is elevated in glioma tissues and cell lines (**A**,**B**) Relative expression of ABCE1 was analyzed by RT-PCR and Western blot analysis in glioma tissues and matched adjacent normal tissues (*n*=38). (**C**,**D**) Relative ABCE1 expression levels were validated by RT-PCR and Western blot analysis in glioma cell lines (U87 and A172) in comparison with the NHA. *P<0.05.

To reveal the effect of ABCE1 on glioma, we detected the expression levels of ABCE1 in 38 pairs of glioma tissues and matched normal brain tissues by RT-PCR and Western blot analysis. The data showed that ABCE1 expression in glioma tissues was significantly higher than that in the normal brain tissues at both mRNA and protein levels (Figure 1A,B). We validated the expression levels of ABCE1 in two glioma cell lines. The results revealed that the mRNA and protein expression of ABCE1 were markedly up-regulated in U87 and A172 cells in comparison with the NHA (Figure 1C,D).

### Down-regulation of ABCE1 inhibits TMZ resistance of glioma cells

**Figure 2:**
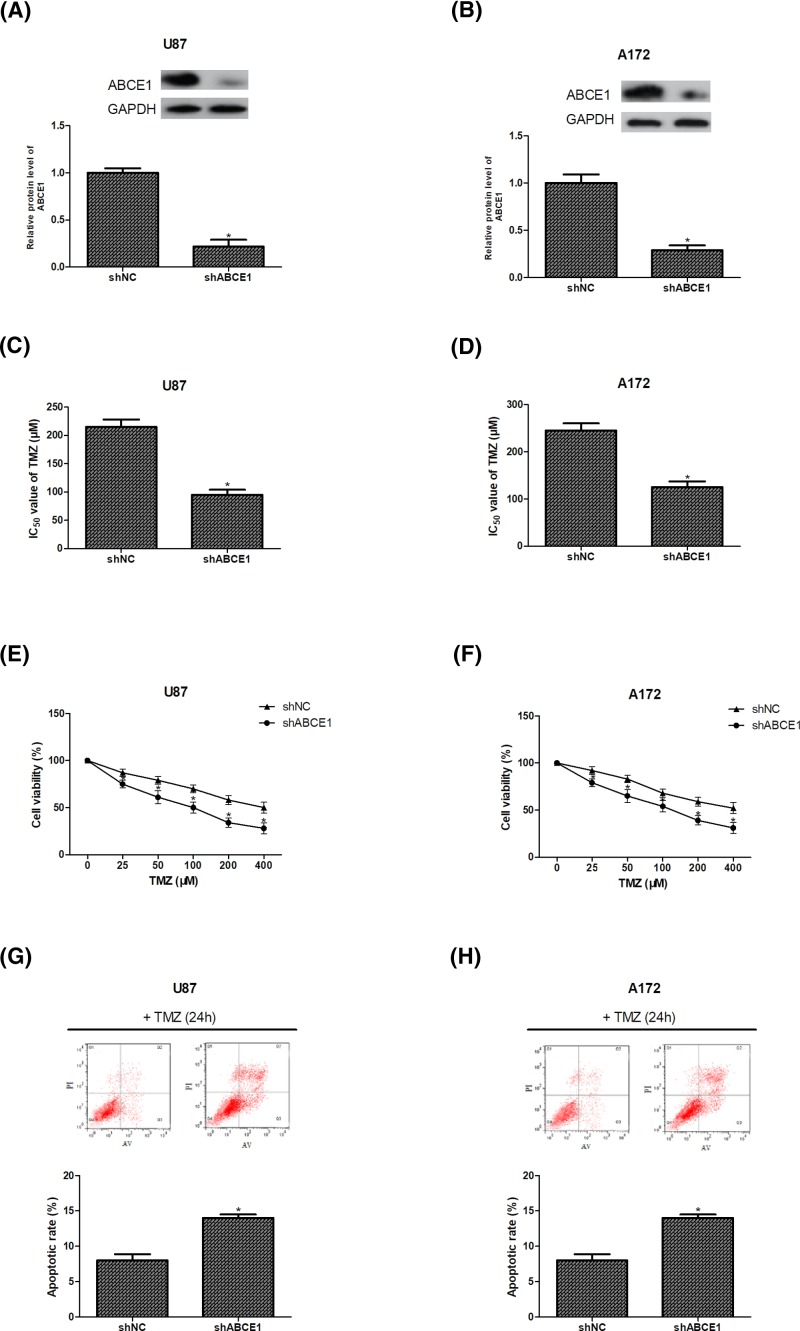
Down-regulation of ABCE1 inhibits TMZ resistance of glioma cells (**A**,**B**) ABCE1 protein levels in U87 and A172 cells were assessed by Western blot analysis after transfection of ABCE1 shRNA (shABCE1) or negative control shRNA (shNC). (**C**–**F**) IC_50_ and cell viability were determined using the CCK-8 assay after treatment with different concentrations of TMZ. (**G**,**H**) The apoptotic rate of U87 and A172 cells was measured by flow cytometry after treatment with TMZ (100 μM). *P<0.05.

To investigate the potential role of ABCE1 in glioma chemoresistance, ABCE1 expression was decreased in U87 and A172 cells by transfection with ABCE1 shRNA. ABCE1 down-regulation was confirmed by Western blot analysis (Figure 2A,B). The sensitivity of shABCE1-transfected U87 and A172 cells to different concentrations of TMZ was evaluated 48 h after drug treatment. The results showed that the IC_50_ value of TMZ was remarkably reduced in ABCE1-knockdown U87 and A172 cells in comparison with the control cells (Figure 2C,D). In addition, the sensitivity to TMZ was significantly enhanced after ABCE1 down-regulation in U87 and A172 cells (Figure 2E,F).

We also performed flow cytometry to detect the effect of ABCE1 down-regulation on glioma cell apoptosis induced by chemotherapy. As shown in Figure 2G,H, knockdown of ABCE1 increased the apoptotic rate of U87 and A172 cells after treatment with the same concentration of TMZ.

### Down-regulation of ABCE1 enhances glioma sensitivity to TMZ *in vivo*

**Figure 3:**
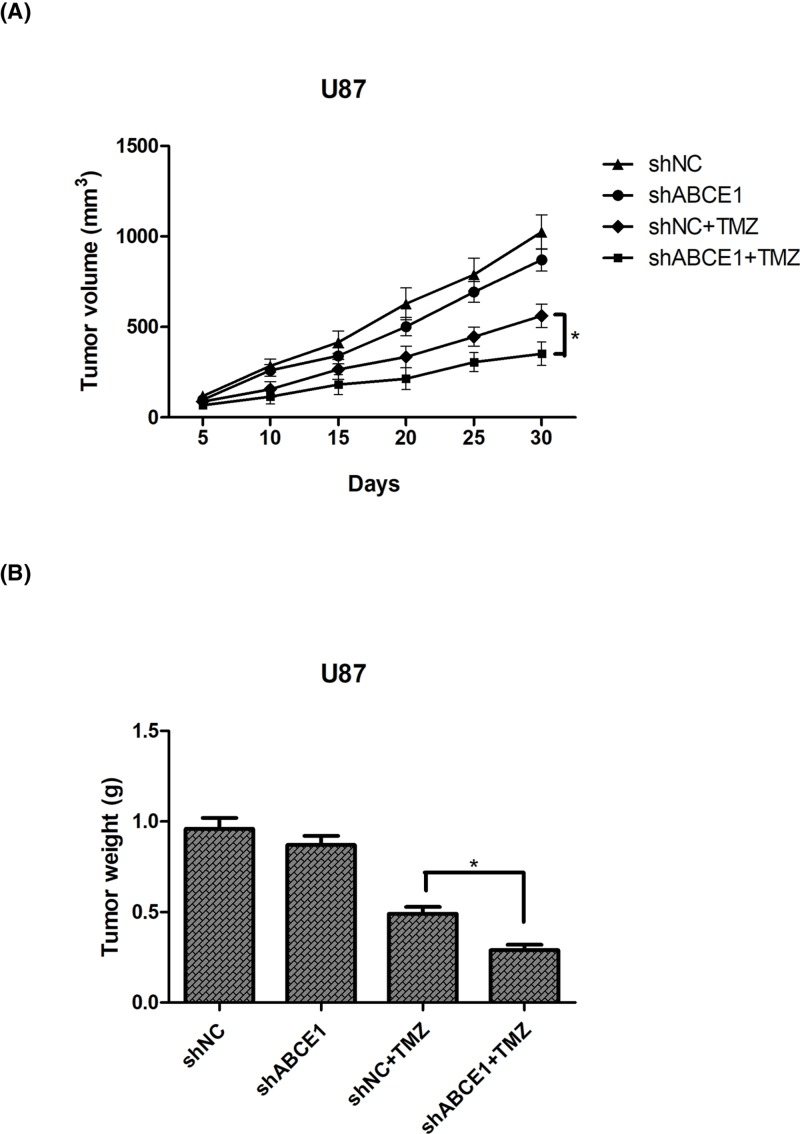
Down-regulation of ABCE1 enhances glioma sensitivity to TMZ *in vivo* (**A**) Growth curves of tumors in mice injected with shABCE1- or shNC-transfected U87 cells, followed by treatment with TMZ. (**B**) The tumor weight was measured 30 days after injection. *P<0.05.

To confirm the above *in vitro* results, we performed xenograft tumor assay. Mice were injected with shABCE1- or shNC-transfected U87 cells. After subcutaneous tumors grew to ∼150 mm^3^, mice were treated with TMZ. As shown in Figure 3A, tumors formed by shABCE1-transfected U87 cells had a slower growth rate in comparison with the control group. Thirty days after injection, tumors were stripped and weighed. The results showed that ABCE1 down-regulation significantly decreased the tumor weight after treatment with TMZ in comparison with the control group (Figure 3B).

### The PI3K/Akt/NF-κB signaling pathway is involved in ABCE1-induced TMZ resistance of glioma cells

**Figure 4:**
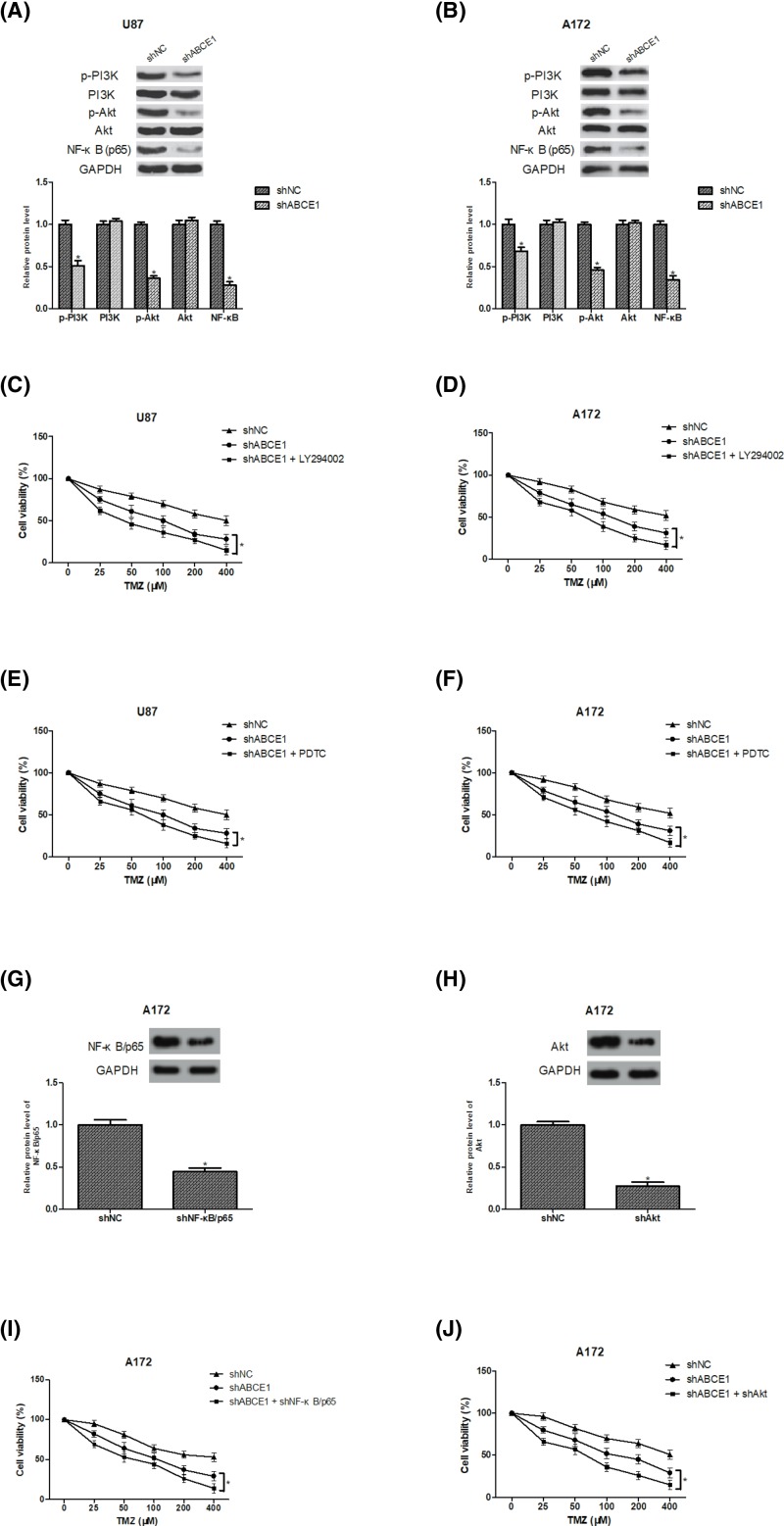
The PI3K/Akt/NF-κB signaling pathway is involved in ABCE1-induced TMZ resistance of glioma cells (**A**,**B**) The protein expression of p-PI3K, PI3K, p-Akt, Akt, and NF-κB was measured by Western blot analysis in U87 and A172 cells. (**C**–**F**) Cell viability was determined using the CCK-8 assay in the presence or absence of LY294002 (20 μM) or PDTC (10 μM). (**G**,**H**) The protein levels of NF-κB and Akt in A172 cells were assessed by Western blot analysis after transfection. (**I**,**J**) Cell viability was determined using the CCK-8 assay after deletion of NF-κB/p65 and Akt. *P<0.05.

Previous studies have revealed that the PI3K/Akt signaling pathway is frequently activated during glioma progression and its downstream target NF-κB is a well-known contributor to chemoresistance [21,22], thus we examined the effect of ABCE1 down-regulation on the PI3K/Akt/NF-κB pathway. The results showed that down-regulation of ABCE1 dramatically decreased the protein expression of p-PI3K, p-Akt, and NF-κB in U87 and A172 cells in comparison with the control cells (Figure 4A,B). To further determine whether ABCE1 down-regulation inhibited TMZ resistance of glioma cells via inactivation of the PI3K/Akt/NF-κB pathway, LY294002 (Akt inhibitor) and PDTC (NF-κB inhibitor) were respectively used after TMZ treatment. The results revealed that the blockade of PI3K/Akt pathway enhanced the inhibitory effect of ABCE1 down-regulation on TMZ resistance in U87 and A172 cells (Figure 4C,D). We obtained similar results after treatment with PDTC (Figure 4E,F). In addition, we found that deletion of NF-κB/p65 and Akt by shRNA also potentiated the inhibitory effect of ABCE1 down-regulation on TMZ resistance in A172 cells (Figure 4G–J).

## Discussion

Glioma, a type of intracranial neoplasm, is characterized by rapid growth and high aggressiveness [23]. So far, TMZ chemotherapy has been established as a standard approach for glioma treatment, but acquired chemoresistance has emerged as a non-negligible obstacle leading to failure in glioma treatment [20,24]. Therefore, focussing on enhancement of the sensitivity to TMZ will be helpful for improvement in glioma treatment.

ABCE1, a member of the ABC family, was originally described as the RNase L inhibitor [25]. Through forming a heterodimer with RNase L, ABCE1 participates in the negative regulation of the 2-5A/RNase L system and thus mediates a wide range of biological functions [26,27]. Recent evidence has shown the new roles of ABCE1 in tumorigenesis. For example, Tian et al. [28] found overexpression of ABCE1 in lung adenocarcinoma cells and the augmentative effect of ABCE1 on tumor growth and metastasis *in vivo*. Huang et al. [29] reported that ABCE1 deletion could induce apoptosis and inhibit proliferation and migration of esophageal cancer cells. In thyroid cancer, ABCE1 was demonstrated to enhance cell viability and invasion *in vitro* [17]. Notably, ABCE1 has also been found involved in the development of chemotherapeutic resistance in cancer cells. For instance, Kara et al. [30] showed that ABCE1 could negatively regulate the sensitivity of lung cancer cells to chemotherapeutic agents. Consistent with the above findings in other types of cancer cells, our study also demonstrated a tumor-promotion role of ABCE1 in glioma by exploration of its expression pattern and biological functions. We found that ABCE1 expression was elevated in glioma tissues and cell lines. Moreover, we observed that ABCE1 down-regulation significantly inhibited TMZ resistance of glioma cells *in vitro* and *in vivo*. Our results suggested a promising therapeutic potential of ABCE1 in improvement of the chemotherapeutic outcome during glioma treatment.

Numerous studies have demonstrated a close association between the PI3K/Akt pathway and glioma progression [31–36]. Akt is a primary regulator of PI3K-initiated signaling and its activation contributes to chemoresistance [37,38]. In addition, one of the downstream substrates of Akt, NF-κB, is an essential initiator of the inflammatory transcription pathway in a diverse range of cancers [39–47]. More importantly, the crucial role of NF-κB has been reported many times in glioma [48–50]. These observations led to our hypothesis that the PI3K/Akt/NF-κB signaling pathway may be involved in ABCE1-induced TMZ resistance of glioma cells. Our study results showed that down-regulation of ABCE1 significantly reduced the protein expression of p-PI3K, p-Akt, and NF-κB in glioma cells. We used inhibitors of the pathway to further confirm our assumption. The results indicated that inhibition of the PI3K/Akt/NF-κB pathway by LY294002 (Akt inhibitor) and PDTC (NF-κB inhibitor) potentiated the suppressive effect of ABCE1 down-regulation on TMZ resistance in glioma cells.

In conclusion, our data showed that ABCE1 was critical in regulation of glioma sensitivity to TMZ and the PI3K/Akt/NF-κB pathway participated in the regulation. Our study presented a possible mechanism underlying glioma resistance to TMZ and proposed a promising strategy for intervention in glioma chemotherapy.

## Abbreviations

ABC: ATP binding cassette
ABCE1: ABC E1
CCK-8: Cell counting kit-8
NF-kB: nuclear factor-kappa B
NHA: normal human astrocyte
TBST: Tris Buffered Saline with Tween-20
TMZ: temozolomide
